# Paediatric Medical Imaging and Radiation Therapy

**DOI:** 10.1002/jmrs.70080

**Published:** 2026-03-08

**Authors:** John Pearn

**Affiliations:** ^1^ Centre for Children's Health Research Queensland Children's Hospital Brisbane Queensland Australia

## Abstract

Every day throughout the world, medical imaging and radiation therapy leads to the diagnosis and potential cure of countless children. The year 2026 commemorates several milestones which have laid the foundations of contemporary best‐practice. World Radiotherapy Awareness Day is a significant international commemoration and a time to reflect on life‐saving and life‐enriching practices which are based on the radiation specialties, and a time to reflect both on their origins and their future potential.

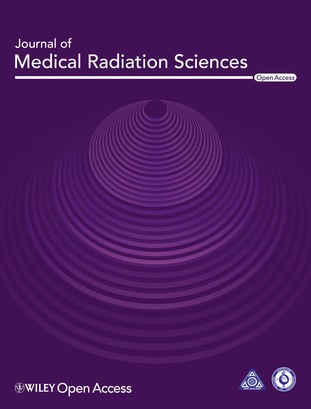

Paediatric medical imaging, like the discovery of antibiotics, changed paediatric care forever.

The year 2026 commemorates several milestones which have laid the foundation for contemporary best‐practice paediatric medical imaging. It is 135 years since Gabriel Lippmann (1845–1921) discovered the reverse piezoelectric electric effect [1]; the basis of ultrasonic diagnosis today [2]. It is 95 years since Paul Dirac (1902–1984), in 1931, postulated the existence of a new type of particle, the anti‐electron, discovered in the next year by Carl David Anderson (1905–1991) and named the positron [3]. The year 2026 also marks the 75th anniversary of the first two papers which described the development of PET scanning to localise brain tumours [4, 5]. It is also the 75th anniversary of Lars Leksell's seminal paper describing his invention of stereotactic radiosurgery [6]. This year is also the Silver Jubilee of the first commercial combination of PET scanning with CT scanning [7].

Today, in their everyday clinical service, radiation therapists, radiographers and sonographers use the fruits of these inventions in a bewildering array of state‐of‐the‐art inventions which range from the Gamma Knife to PET‐MRI scanners and Photon Counting CT scanners.

Gone are the days (e.g., in the 1930s) when a hospital gardener could function as the diagnostic radiographer [8]. Modern day radiographers, sonographers and radiation therapists are University trained. Their clinical lives range from advanced research projects to patient‐care sociology.

Since 1953, when the first oncology patient was treated using a linear accelerator, so much has been achieved—cures, symptom relief and prolonged survival. Advocacy to raise awareness among the public about the crucial role of radiotherapy in cancer patients led, on 7 September 2025, to the launch of World Radiotherapy Awareness Day, a significant International commemoration ‘which is positioned to become a powerful advocate for the life‐saving and life‐enriching potential of radiotherapy across the world’ [9]. The choice of 7 September as the annual World Radiotherapy Awareness Day is significant as that is the date, in 1953, when the first patient was treated by radiation generated in a linear accelerator.

In the history of science, revolutionary discoveries have always produced unexpected consequences. The history of radiation medicine is no exception. In the first decade which followed Röntgen's 1895 discovery of x‐rays, there developed an increasing understanding of the risks of radiation. By the end of the first decade after Röntgen's discovery, acute radiation burns and chronic radiation dermatitis were well‐recognised as risks to both patients and nuclear medicine specialists [10]. The risks to radiologists and radiographers, particularly the development of painful keratoses and skin cancers of the hands, were increasingly described by pioneers in many countries [11].

It took several decades for post radiation exposure and the risk of general malignancy to be identified. Many ‘ray attendants’ died prematurely in the first decades of the 20th century before the risk of general malignancy was identified [12]. It was not until 1957 that the link between radiation exposure and the later development of cancers was proven by Dr. Court‐Brown and Dr. Richard Doll, the latter the Oxford epidemiologist who also established the causal link between smoking and lung cancer [13]. These workers further demonstrated that there was no safe ‘threshold dose’ of radiation; and that even for low cumulative doses, the risk of later leukaemia bore a simple proportional relationship to the life‐time cumulative dose of radiation [14]. The practical risk was much greater in the early years of x‐ray use; and particularly affected those who cared for children. Altruistic radiographers and radiologists often held a struggling or fearful child's limb during exposure to the x‐ray beam. It required the experience and research of future decades for society to acknowledge the altruistic service of those singular pioneers. The evidence derived from longitudinal safety studies has led to the safe, evidence‐based safety practices which are the norm today [15].

Another unexpected consequence of the discovery of x‐rays was a threat to the established order of bedside diagnosis. This was a very human and psychological consequence. For the first time in the living patient, the radiologist's report was in one sense an audit of the clinician's bedside diagnosis. In some cases this was threatening to the paternalistic dominance of the clinician, especially that of surgeons. The radiologist's report was written and was usually exact. If a clinician had been wrong, the x‐ray report remained as a long‐lasting witness of such clinical error. In 1897, at the 48th Annual Meeting of the American Medical Association, Professor Leonard acknowledged that the ‘tangible shadows’ of x‐rays removed a source of common error of the attendant clinician but:

It is worse than useless to suppose that a new method of forming mental pictures, no matter how startling or radical, can equal in accuracy or value that which the science of medical diagnosis has already taught us to form with well‐nigh infallible precision [16].

This arrogant and paternalistic approach contrasted with the humility of pioneering radiologists and the medical radiation professionals who supported them [17].

A second consequence of the invention of radiographic and later sonographic diagnosis was, and remains, the tendency to bypass the clinician's discipline and rigour of formulating a differential diagnosis. Pivotal to best‐practice clinical medicine in the 21st century is the concept of differential diagnosis. This comprises a six‐link chain comprising history‐taking; clinical examination; differential diagnosis; test investigations of which radiological or sonographic studies are a crucial part; provisional or definitive diagnosis; and finally, management. This paradigm evolved to its present form, if not in name, in Western medicine and its derivatives, with the publication of Osler's *The Principles and Practice of Medicine*, in 1892 [18].

It is so easy to order radiological or sonographic tests as a primary ‘diagnostic fishing expedition’, that the gold standard of formulating a differential diagnosis can be bypassed. This has the consequence that, occasionally, a definitive diagnosis is delayed or worse, missed and only revealed at autopsy.

Radiographers, sonographers and radiologists constantly make decisions about the utility of different investigations, decisions which are based on the probability of a positive diagnosis. A recent example in this *Journal*, reporting a research study of investigating the diagnostic utility of spinal ultrasound in neonates with a simple sacrum dimple, demonstrated a very low incidence (1.04%) of spinal dysraphism [19]. Decision making about the optimal tests to undertake is best achieved by close cooperation between clinicians and those actually performing the diagnostic or therapeutic interventions of all patients, not only those caring for children. That is the message of the proud chronology of radiation science in the service of healthcare.

## Conflicts of Interest

The author declares no conflicts of interest.

## Data Availability

The author has nothing to report.
